# “The DEA would come in and destroy you”: a qualitative study of fear and unintended consequences among opioid prescribers in WV

**DOI:** 10.1186/s13011-022-00447-5

**Published:** 2022-03-10

**Authors:** Cara L. Sedney, Treah Haggerty, Patricia Dekeseredy, Divine Nwafor, Martina Angela Caretta, Henry H. Brownstein, Robin A. Pollini

**Affiliations:** 1 Department of Neurosurgery, Rockefeller Neuroscience Institute, West Virginia University, 1 Medical Center Drive, PO Box 9183, Morgantown, WV 26506 USA; 2 Department of Family Medicine, West Virginia University, Morgantown, WV USA; 3 Department of Neuroscience, West Virginia University, Morgantown, WV USA; 4 Human Geography Department, Lund University, Lund, Sweden; 5 Sociology and Anthropology, West Virginia University, Morgantown, WV USA; 6 Departments of Behavioral Medicine and Psychiatry, Department of Epidemiology and Biostatistics, West Virginia University, Morgantown, WV USA

**Keywords:** Opioids, Pain medication, Legislation, Chronic pain

## Abstract

**Background:**

West Virginia has one of the highest rates of opioid overdose related deaths and is known as the epicenter of the opioid crisis in the United States. In an effort to reduce opioid-related harms, SB 273 was signed in 2018, and aimed to restrict opioid prescribing in West Virginia. SB 273 was enacted during a time when physician arrests and convictions had been increasing for years and were becoming more prevalent and more publicized. This study aims to better understand the impact of the legislation on patients and providers.

**Methods:**

Twenty semi-structured interviews were conducted with opioid-prescribing primary care physicians and specialists practicing throughout West Virginia.

**Results:**

Four themes emerged, 1. Fear of disciplinary action, 2. Exacerbation of opioid prescribing fear due to restrictive legislation, 3. Care shifts and treatment gaps, and 4. Conversion to illicit substances. The clinicians recognized the harms of inappropriate prescribing and how this could affect their patients. Decreases in opioid prescribing were already occurring prior to the law implementation. Disciplinary actions against opioid prescribers resulted in prescriber fear, which was then exacerbated by SB 273 and contributed to shifts in care that led to forced tapering and opioid under-prescribing. Providers felt that taking on patients who legitimately required opioids could jeopardize their career.

**Conclusion:**

A holistic and patient-centered approach should be taken by legislative and disciplinary bodies to ensure patients are not abandoned when disciplinary actions are taken against prescribers or new legislation is passed.

## Introduction

The United States faces a nationwide public health crisis initially thought to be fueled by over-prescription of opioid pain relievers [1]. The rise in opioid overdose-related deaths can be outlined in three distinct waves; first involving prescription opioids, then involving heroin, and currently involving illicitly manufactured fentanyl [2–5]. The Centers for Disease Control and Prevention (CDC) in 2017 estimated that roughly 2.4 million Americans suffered from substance use disorders related to prescription opioid pain relievers and illicit opioids like heroin and fentanyl. From 1999 to 2018, an estimated 450,000 Americans died from overdoses involving opioid pain relievers and illicit opioids [6, 7]. Furthermore, the total economic burden of opioid misuse costs the United States approximately $78.5 billion dollars annually, including healthcare cost, lost productivity, and opioid use disorder treatment [8].

Subsequent laws enacted by state and federal governmental bodies contributed to a rapid decline in opioid prescribing [9]. In the United States, opioid prescriptions fell by 54% from 2012 to 2017 [10]. The decline in opioid prescribing in the United States has been applauded as a positive sign towards addressing the opioid epidemic, although increasingly, these reductions are recognized to have unintended consequences [11, 12]. Additionally, the reasons and motivations for these changes warrant examination, particularly because of inconsistent effects of legislative efforts on opioid prescribing, where the anticipated effects of law do not bear out in the real-world consequences [13], which raises the possibility of other drivers behind physician behavior change, such as fear [14]. Physicians have been shown to alter their behavior in relation to lawsuits and other external motivators [15]. Thus, studies assessing whether the rapid decrease in opioid prescribing stems from drivers aside from the legislative restrictions are needed.

**Fig. 1:**
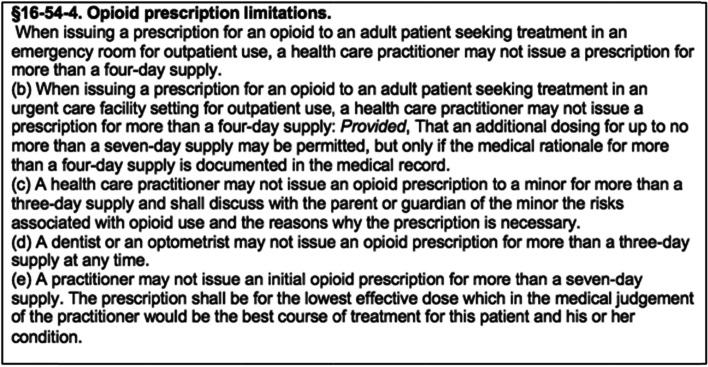
Prescription limitation language in SB273 (Opioid Reduction Act) [19]

West Virginia (WV) has one of the highest rates of opioid overdose related deaths, and has been regarded as the epicenter of the opioid crisis in the United States [16]. Several factors contribute to the high incidence of opioid-related harms in WV; these include wealth inequalities, economic depression, low educational attainment, and high rates of opioid prescription dating back to the mid-90 s [17]. Opioid-related harms and increasing attention to the opioid crisis led to the passage of Senate Bill 273 (SB 273), which was signed on March 27, 2018, in an effort to limit opioid prescribing in WV. The law contains specific limits for initial and subsequent opioid prescriptions but exempts cancer patients, patients in hospice care, palliative care, residents of long term care facilities, patients receiving treatment for substance use disorder, and patients receiving on-going opioid treatment as of January 1, 2018 [18]. See Fig. 1 for prescription limiting language.

In our previous work assessing changes in opioid prescribing related to SB 273, we noted that overall prescription volume trended down during the 64 weeks prior and following law implementation, without an accompanying decrease in day’s supply or first time opioid prescriptions, even though the law itself focused on limiting initial prescriptions [13]. We sought to understand these trends in this subsequent study through in-depth qualitative interviews with stakeholders, including opioid prescribers, in WV. Our hypothesis was that stakeholders may have unique perspectives on why this occurred that would be inaccessible through only quantitative data.

## Methods

For our study of SB 273, we designed a sequential explanatory mixed methods study in which qualitative interviews were used to provide insights into our earlier quantitative assessment of prescription rates [13]. To address the changes in prescribing practices noted in that study, we conducted a qualitative analysis of responses to the interview using content analysis.

### Participants

We recruited a purposive sample of opioid prescribers between the months of March and October 2020. Inclusion criteria were: 1) licensed physician or dentist in the State of WV authorized to prescribe opioid medications (i.e., had a Drug Enforcement Administration [DEA] license) and 2) can read, write and speak English (assessed during informed consent procedure). Participants were recruited through the WV Practice Based Research Network (WVPBRN). The WVPBRN is a group of primary care clinicians and practices across the state of WV partnered with West Virginia University as a research entity [20]. Participants were selected, employing a maximum variation strategy [21], to ensure diversity in age, gender, urban/rural demographics, practice type, and race/ethnicity. Participant specialty was chosen to reflect specialty-specific contributions to the overall opioid prescription number in the state based upon prescription drug monitoring program (PDMP) data with the highest-prescribing specialties represented in proportion to prescribing amount. Potential participants were emailed an introduction to the study and requested to participate. For the primary care physicians, 10 consented and completed the interview, 13 providers were contacted but did not respond, and one provider was contacted and declined. For specialist physicians, 10 consented and completed the interview, one provider was contacted but did not respond, and one additional was contacted and declined. All participants provided written informed consent prior to data collection relating to reasons for the study and all study protocols were approved by the West Virginia University Institutional Review Board protocol # 1,908,659,237.

### Data collection

The 20 participants completed a one-time 30 to 60 min semi-structured interview by telephone or in person. All the interviews were conducted by co-investigator PD, a research nurse trained in qualitative research, and the interview was digitally audio recorded. The semi-structured interview guide consisted of open-ended questions generated by review of our previously documented quantitative opioid prescribing data trends in WV [13]. The interview began with a general discussion of the participant’s specialty, career, and time in WV. Member checking [22] was conducted during interviews to verify accuracy with participants through clarifying questions..

Interview questions focused on.

1. Background on their medical practice
2. Experience with prescribed opioids
3. Legislation and rules impacted opioid prescribing
4. Understanding of SB 273 and impacts to practice
5. Changes in prescribing practices
6. Impact on patient care

Participants received a $30 gift card following participation in the study. Interviews were professionally transcribed verbatim and verified by a co-investigator (PD). Recruitment continued until data saturation was achieved [23, 24].

### Data analysis

The methodological orientation underpinning this study is content analysis. Three co-investigators (CS, PD, TH) generated the code book inductively after preliminary review of transcripts [25]. The three investigators systematically read all interviews and iteratively and inductively generated codes. Memo-writing and group discussion by CS, PD, and TH was utilized to identify and expand parent codes and subcodes, which were then solidified and assessed for validity by MAC and RAP [22]. Codes were entered in NVivo 12 – qualitative data analysis software – together with the interviews transcripts to generate summaries of themes by MAC and RAP. An audit trail was maintained throughout data collection and analysis process. Methodological rigor was enhanced by incorporating a multidisciplinary research team. Methodological triangulation between participants as well as investigator triangulation was used as described by Carter and colleagues for qualitative research [26]. We additionally triangulated our qualitative results with our previously published autoregressive integrated moving average (ARIMA) analysis of prescribing data for the same timeframe from the West Virginia Board of Pharmacy [13] in a sequential explanatory fashion. Reporting was cross checked with both COnsolidated criteria for REporting Qualitative research (COREQ) and Standards for Reporting Qualitative Research (SRQR) checklists for qualitative research.

## Results

### Participant characteristics

Twenty individuals were interviewed consisting of 12 male and 8 female prescribers. Participants included 10 primary care physicians (PCPs) (primary care physician providing acute and chronic care, health education and preventative care) and 10 specialists (SP). The specialist prescriber group included three orthopedic surgeons, a pain specialist, rehabilitation specialist, general surgeon, anesthesiologist, emergency medicine physician, palliative care physician, and dentist. Practice locations were geographically distributed across the state (6 northern and 4 southern counties; 4 counties representing the 4 main metropolitan areas and 6 representing more rural areas) and represented 10 of the 55 counties in West Virginia. The PCPs’ years of practice ranged from 6 to 30 years with an average of 15.3. The specialists’ years of practice ranged from 10 to 31 years with an average of 17.5 years. Based upon the qualitative analysis, four themes emerged. These were: 1. Fear of disciplinary action, 2. Exacerbation of opioid prescribing fear due to restrictive legislation, 3. Resulting care shifts and treatment gaps, and 4. Conversion to illicit substances.

### Findings

#### Fear of disciplinary action

**Fig. 2:**
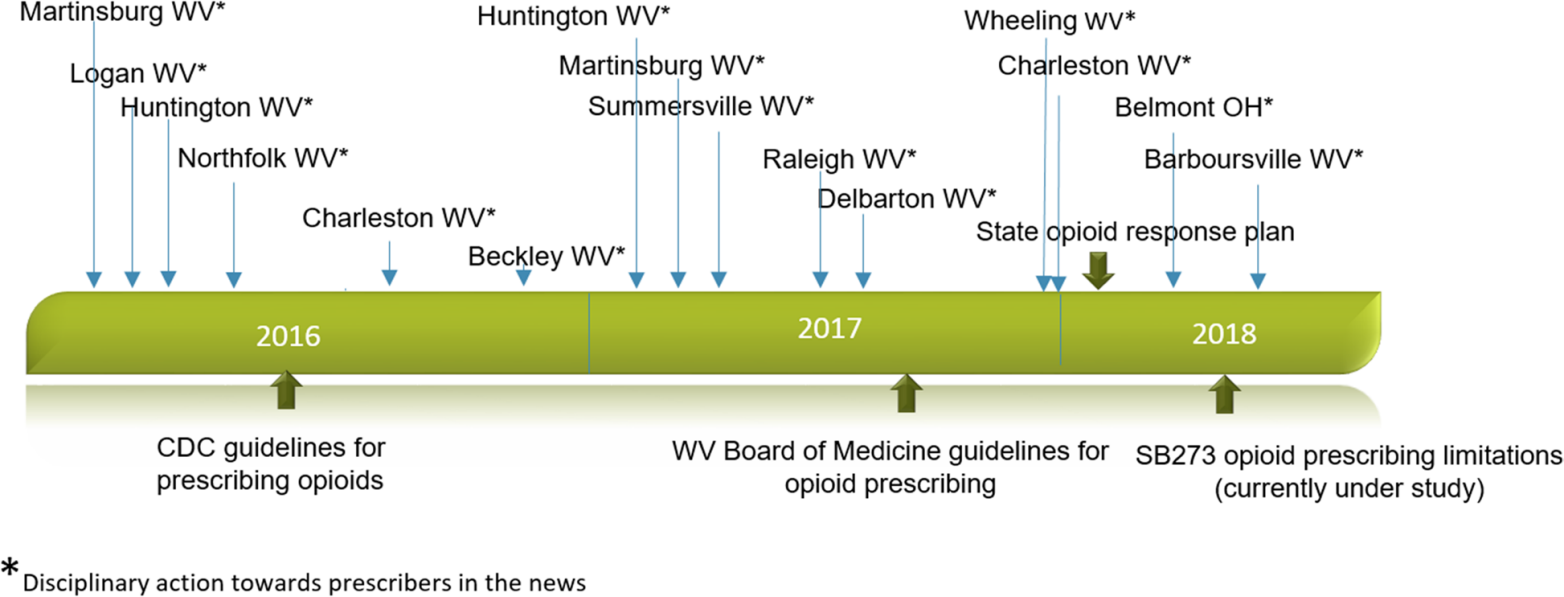
Timeline of Opioid Prescribing Influences

SB 273 was enacted during a time when physician arrests and convictions had been increasing for years and were becoming more prevalent and more publicized. Figure 2 details some of the many investigations and disciplinary actions against physicians in the time period leading up to the enactment of SB 273, along with other societal events pertaining to opioid prescribing. The events described by participants often occurred before SB 273, resulting in behavioral change in prescribing prior to the implementation of the law. With respect to disciplinary action, physicians under investigation in the years prior to law implementation had their practice interrupted, including having records confiscated and offices closed, which other physicians often became aware of anecdotally through community contacts, patients, or other providers. Often such investigations and charge filings were publicized on a local or state level, broadening the sphere of impact of the actions -these included lay news media publications, board of medicine announcements of disciplinary action, etc. Physicians were portrayed as criminals in the lay media, as in these quotes from a U.S. Attorney: “Home-grown drug dealers hidden behind the veil of a doctor’s lab coat, a medical degree, and a prescription pad, are every bit as bad as the heroin dealers that flood into WV” [27] and WV Assistant Attorney General: “To the doctors, pharmacists, and other medical professionals engaged in this egregious criminal behavior across Appalachia and our country, the data in our possession allows us to see you and see you clearly, no matter where you are… and if you behave like a drug dealer, we will find you and ensure that the American justice system treats you like the drug dealer you are” [28]. Determination of guilt could be years after the initial investigation, and importantly, some physicians were never charged or found guilty after having their practice interrupted with lengthy investigations. Most of these criminal proceedings occurred before SB 273 was implemented.

News coverage of such disciplinary actions against physicians included some legal and allowable practices in primary care as evidence of guilt. Among these were operating a cash-based practice, not accepting insurance, charges of “$240 for an initial appointment and at least $160 for each subsequent appointment”, not requiring referrals, receiving patient calls on a personal cell phone, and not having special training in pain management to prescribe opioids [27].

Participants referenced multiple instances of known physicians, either in their own community or people they heard about through media or news stories, who suffered disciplinary action for opioid prescribing in the years prior to SB 273. Some prescribers against whom disciplinary action was taken were acknowledged to be practicing inappropriately and disciplinary action was felt to be warranted. However, disciplinary action against these outlying prescribers resulted in fear amongst other prescribers and affected their prescribing habits, overpowering other considerations in decisions regarding prescribing. This fear was expressed by multiple participants and consequences included licensure revocation or criminal penalties related to opioid prescribing which the prescriber feared would be seen as excessive or inappropriate, even if they deemed it medically necessary. The resulting practice changes moderated opioid prescribing prior to implementation of SB 273. Licensing boards, the Drug Enforcement Administration, and local law enforcement were seen as enforcers. As one participant noted:

“…the fear of having your license taken away or the fear of imprisonment, or you’ve been burned by the DEA as someone who has been negligent, that will take over.” (PCP, female, 15 years of practice)

These enforcers were alerted by pharmacists or the Board of Medicine that a prescribers’ practices were concerning, creating a conflicted environment between the two groups as prescribers felt like they were being policed by dispensers in their community and their medical judgement was being questioned.

“[M]ost of us noticed that whenever all of the pill mills got shut down prior to the law, it was because pharmacists were reporting the doctors who were writing too many prescriptions to the DEA and then the DEA would come and destroy you.” (PCP, male, 11 years of practice)

These prescribers detailed that some providers caught up in disciplinary action were viewed as “good doctors” or were practicing within what they perceived to be accepted norms of patient care. In some cases, the physicians were never charged with any crimes, which the participants understood to be evidence that they were practicing appropriately, but those prescribers could not recover after their practice had been shut down, records confiscated, or had poor publicity in the media. This resulted in a reluctance to prescribe opioids even if pain medication was perceived to be needed by patients. Another participant noted that even being “outside of the norm” of opioid prescribing based upon labelling or assumptions rather than practice which was technically unlawful was seen as high risk for disciplinary action:

“[It] really started to scare a lot … of providers into feeling that it wasn't worth the risk to continue to prescribe for fear of being labeled as an over prescriber or being outside of the norm or, you know, the potential liability that goes along with it.“ (PCP, male, 14 years of practice)

These actions created an atmosphere of fear amongst prescribers which, according to one prescriber, preceded restrictive prescribing laws in WV and rendered these subsequent actions unnecessary.

“They were coming in and busting a lot of docs and then making it so… that we didn't need that law to be afraid.” (PCP, female, 11 years of practice)

This temporal association (reactionary fear of prescribing opioid medications to patients based upon disciplinary action before SB 273) correlates to our previously published quantitative findings of opioid prescribing trends in WV, which documented consistent declines in opioid prescribing that preceded the implementation of the 2018 law [13]. That is, providers were already fearful of publicity or practice interruption from baseless disciplinary actions even before the passage of the bill, and therefore the threat alone interfered with their judgement and ability to make medical decisions about how to treat their patients.

#### Opioid legislation exacerbated prescriber fear

In addition to disciplinary actions against prescribers, numerous other instructions, guidelines, and regulations impacted the prescribing of opioid medication leading up to the enactment of SB 273 (Fig. 2). The CDC created prescribing guidelines for opioid medication in March 2016 [29], and the WV Board of Medicine also created opioid guidelines in 2017 [30], both with the focus of curtailing inappropriate prescribing. The state of WV also created an “Opioid Response Plan” in January 2018 [31] to handle what was perceived as inappropriate prescribing of opioids. As a result, nearly all participants in our study noted that prescribing of opioids, both their own habits and what they observed in their community and amongst their partners, evolved towards moderation over time as knowledge of opioid-related harms increased. This is supported by our earlier quantitative work assessing opioid prescribing practices in the 64 weeks before and after SB 273 was implemented, demonstrating a decreasing trend throughout the entire period before the law [13]. Furthermore, our assessment of key requirements of SB 273, such as duration of opioid prescriptions (4–7 days for new prescriptions; 30 days for established prescriptions), demonstrated no change in relation to SB 273 – however, over the duration of our study, the days’ supply of medication decreased from 13.9 days to 7.9 and 7.3 days prior to law signing and enactment respectively, making current clinical practice at the time of law signing and enactment already in line with the 7 day prescribing limit of new opioid prescriptions for SB 273 [13].

SB 273, while implemented to address opioid over-prescribing, was viewed as exacerbating the fear already being experienced by prescribers, and for which prescribers had already changed their practice. As a result, many participants felt that the law had minimal impact because most prescribers had already curtailed opioid prescribing as a result of CDC guidelines or other influences. However, participants noted that this law prompted further, more extreme changes in practice amongst physicians who otherwise felt they were practicing appropriately, such as cessation of all opioid prescribing, particularly amongst prescribers that primarily dealt with chronic conditions. The prevailing view of participants was that all of the outlying or “irresponsible” prescribers had already been dealt with prior to SB 273. However, the law exacerbated the pre-existing fear of disciplinary action and led many prescribers to further curtail opioid prescriptions, and in some cases, refuse to prescribe any controlled substance including medications like gabapentin (Schedule 5).

“[SB 273] ended up affecting mostly the responsible prescribers of opioids. The people who were already doing due diligence ended up being the ones who were concerned about the law because all the irresponsible prescribers I think were already getting obliterated.” (PCP, male, 11 years of practice)

Participants expressed an understanding of the roles of different regulatory and enforcement bodies and the difference between state and federal laws, but the overall effect seemed one of cumulative risk/fear from various quarters which led to reactionary changes in practice. A “chilling effect” instigated by fear of disciplinary action and exacerbated by SB 273 created a dearth of prescribers willing to provide opioid prescriptions within entire communities as it was considered to be increasingly risk-laden as a result of codifying restrictive prescribing practices into law. This occurred even as the need for opioid medication was recognized within the prescriber community. The enormity of the need was recognized by prescribers even as they did not feel capable of meeting it themselves:

“Almost all prescribers in WV cut it back, a few that still do it [prescribe], really do so at great risk to themselves. I think the law that occurred in 2018, really, if anything shifted us to a place where there's not enough opioid prescribing for many painful conditions that aren't treatable with other means.” (PCP, male, 11 years of practice)

The change was noted among prescribers across practice types and locations – but was more pronounced in prescribers who did more chronic opioid prescribing, such as general practice and pain management, and included patients with cancer-related pain or those which were taking opioids prior to January 1, 2018, both populations which were specifically excluded by the law.

“There was a dramatic reduction in the number of physicians in the area who were writing anything controlled. It didn't matter if it was a schedule 2 or a schedule 5. They just said, "No, I'm not going to do this. I'm not putting my license on the line." (PCP, female, 10 years of practice)

It is important to note that, as noted above, the law itself excluded patients who were taking chronic opioids prior to law signing (just three months prior to law enactment), therefore the prescriber fear in reaction to the law may have been largely unfounded, especially with regard to chronic pain patients, but the real world effects were no less pronounced.

#### Care shifts and treatment gaps of chronic opioid management resulted from disciplinary actions and legislation

The fear created by disciplinary actions against other prescribers prior to SB 273 and then exacerbated by SB 273 led to refusal by some prescribers to continue prescribing opioid medications; this created care shifts and treatment gaps, particularly for chronic pain patients, around the time SB 273 was implemented. Care gaps for acute pain conditions were not described. This, again, is supported by our prior quantitative findings [13]. While first time opioid prescriptions and days’ supply, which were the specific focus of SB 273, did not change in relation to the law, overall prescription numbers decreased, demonstrating a differential impact on continuing opioid medications. Participants described these care shifts and treatment gaps as negatively affecting patient care, as patients could not access continued opioid medication for their chronic conditions. Prescribers detailed a chaotic in-flooding of patients left with no prescriber for their opioid medications, and this care shifted to remaining physicians in the community. These patients either shifted to other primary care physicians, specialty pain physicians, or were in effect forcibly tapered.

“Every time a physician is raided or arrested, we get several hundred referrals of those patients who are, either appropriately, or inappropriately on opioids that we have to sort out.” (Specialist, male, over 30 years of practice)

Treatment gaps in chronic opioid management represented de facto patient abandonment amongst prescribers who stopped prescribing due to fear of disciplinary action in light of the new legislation. Some general practice physicians refused to treat chronic pain completely. Often these patients were in effect forcibly tapered or transferred to specialty pain clinics in faraway locations. Participants describe patients attempting to change physicians after their own physician refused to continue writing chronic opioids with only a week’s worth of medications or enough medication to get them to the date of the specialist clinic visit. Specialists noted an increase in patient referrals requesting to take over long-term opioid management for their chronic pain after their primary care physicians refused upon passage of SB 273. Primary care participants noted that these patients were a stretch on already tenuous rural clinic capacity due to the number of patients and the level of management required.

“When these prescribers were shut down by the, either Board of Medicine or the DEA, we had to pick up the pieces as other local physicians-- well, we would get an influx of patients from these doctors each time” (PCP, male, 30 years of practice)

#### Conversion to illicit substances

The perception of many prescriber participants was that patients who were left without opioids, both from provider refusal due to fear of disciplinary action and later restrictive opioid legislation, or from disciplinary action against the provider causing abrupt lack of care, transitioned to illicit substances such as heroin. One participant reported direct experience with patients as they later presented in a clinical capacity asking for help with their substance use disorder, and another physician reported that he obtained his X-waiver purely to meet the patient need of those whom had been forcibly tapered off of chronic opioid medication.

“[T]hey show up at my door a year later, using heroin for a year… saying, ‘I need help.’ And I said, ‘Well, what happened?’ ‘Well, I was getting a legitimate prescription, and then they stopped…’ And it's definitely, honestly, it's a lot more now than what it was before this legislation.” (PCP, male, 14 years of practice)

Participants noted that these patients often directly related this transition to changes in the availability of prescribed opioids for chronic pain. The participants felt that these care gaps and unintended consequences were created by disciplinary actions taken against physicians prior to SB273 without considering the impact on the patients receiving chronic opioid medication, but were exacerbated by the law.

“That was a big problem and a big oversight on behalf of law enforcement and the physician community… We didn’t have a good plan when we got these doctors down. We didn’t- we didn’t go in and find all these patients and… pick up the pieces from these patients. So that we could appropriately taper them and in a way that managed … their withdrawal symptoms and their dependency without them turning to the illicit market. And I think that’s a big source of a lot of our problems.” (PCP, male, 30 years of practice)

Participants recognized that these unintended effects were understudied but have had potentially significant effects on communities in WV.

## Discussion

Opioid over-prescribing is widely recognized as playing a critical role in the genesis of the opioid crisis. However, while the clinicians we interviewed recognized the harms of inappropriate prescribing and acknowledged that this affected their patients, they felt that decreases in opioid prescribing were already occurring prior to the implementation of SB 273. A decrease in opioid prescribing prior to law implementation was also seen in our quantitative data analysis [13]. This is consistent with other quantitative studies, as Sutherland and colleagues note a decrease in opioid dispensing after surgery related to the 2016 CDC guidelines on opioid prescribing [32], and Ranapurwala and colleagues noted a similar decline after state-level Board of Medicine opioid prescribing limitation policy [33]. In our sample, fear of disciplinary action was frequently cited as a motivator to curtail or completely cease opioid prescribing both before SB 273 and worsening as a result of SB 273.

The possibility of other factors besides restrictive opioid prescribing laws driving the decrease of opioid-related prescribing is supported in the marginal impact of such laws in various analyses. In our ARIMA analysis of SB 273, we documented a decrease of overall opioid prescriptions without an accompanying decrease in first time prescriptions or days’ supply, even though they were the main foci of SB 273 [13]. Minimal or inconsistent impact was also seen by a variety of other groups employing a variety of other quantitative analyses for similar laws in other states [34–36]. Prior to these relatively recent legislative attempts, prescription drug monitoring programs were initiated with the same goal. Several studies have reported reducing availability of controlled substances with physician utilization of prescription drug monitoring programs [37–39]; however again the effects varied by state [40].

The clinicians we interviewed describe a reactionary process whereby despite the good intent of such restrictive laws, patients who need opioid medications for pain management may not get them due to prescriber fear of disciplinary action. This contributed to patient abandonment, in situations where physicians had already curtailed any inappropriate prescribing prior to implementation SB 273. The downstream effects of the under-prescribing or sudden reduction in access to opioid treatment due to prescriber fear of disciplinary action in WV, both before SB 273 and exacerbated by it, may have driven unintended consequences. Such unintended consequences are the subject of recent study, with links related to undertreated pain, increasing experienced stigma amongst pain patients, [11] and increased overdose risk amongst patients [12]. On an economic level, some researchers estimate that undertreatment of pain may lead to loss of productivity costing the United Sates as much as $299 billion annually [41]. Our participants related care gaps and forced tapering as a result of SB 273, similar to that reported by Dickson-Gomez and colleagues as unintended consequences of PDMP implementation in various states [42]. The idea that such supply-side restrictions on prescribed opioids may drive illicit use and opioid related deaths has also been explored and supported in a further quantitative analysis by Kim [43], as well as in further related work on methamphetamine markets [44].

There is little existing literature regarding the effect of fear of disciplinary actions upon prescriber behavior, but it may be understood in the context of “defensive medicine” which is defined as “a physician’s deviation from what is considered to be good practice to prevent complaints” [45]. Qualitative analysis of defensive medicine practices has noted that “positive defensive medicine changes” such as increased diagnostic work, referrals, or time spent counselling or consenting patients is not related to a specific enforcement event, but that “negative defensive medicine changes” such as “withdrawal from the doctor-patient relationship and particular fields of practice” were related to specific complaints or enforcement events [46]. Our data supports this assertion since the prescribers interviewed in this study stated that litigation or criminal actions against physicians in high-profiled cases led to some prescribers change their practice in the years prior to SB 273, and later to stop opioid prescription altogether when restrictions were codified into law.

Like other qualitative interview-based studies, desirability bias may play a role in our physician interviews. Doctors often referred to others instead of themselves, which may have misrepresented their own thoughts or experiences. In particular, the influence of fear on practice pattern changes was often referenced in terms of “other providers” but may in fact be a reflection of the influence of fear on the participant physician’s own practices. When asked specifically about their own practices, both before SB273 and resulting from SB273, the overwhelming majority noted that their practice became more conservative with regard to opioids over time resulting from a variety of influences including the 2016 CDC guidelines. Curtailing of prescription opioids overall, however, was more commonly described in terms of “other providers.” This dichotomy may be due to the negative impacts of the decision to not prescribe, which the participants recognized, or to a recognition by some of the participants that they were, in fact, previously practicing outside accepted norms. Further exploration of prescribers’ own practices versus their perception of others’ practices may be helpful.

Some other limitations exist in this study. We utilized a variety of techniques to ensure validity and reliability of our results [22]. Our number of interviews (*N* = 20) enabled both diversity of narrative and also theme saturation, while our sampling plan enabled a broad assessment across prescriber demographics, specialty, location, and practice type. Although we recruited with an eye towards diversity and achieved thematic saturation, the findings do not apply to all providers in WV and may not apply to other states with different demographics.

The fear of prescribers surrounding opioid prescriptions, which originated from disciplinary actions and was later exacerbated by SB 273, highlights the importance of successful dissemination of new laws impacting the practice of medicine, since the wording of the law may not reflect the real world impact if the nuances and exclusions within the law are not well-understood by providers. Furthermore, particularly with respect to laws addressing medical topics for which care abandonment may be of concern due to liability issues, safeguards to ensure continuity of care and avoid patient abandonment may be key. This might include special allowances for tapering or requiring joint decision making. Coupling restrictive opioid prescribing laws with mechanisms to increase the capacity of pain or addiction treatment may be helpful. Because of the consistent tension between what prescribers felt they were allowed to prescribe to patients against the actual medical needs of the patients, an exemption based upon medical judgement, which is present in some restrictive opioid prescribing laws but not in SB273, may be helpful. Lastly, because prescription opioids are no longer thought to drive the opioid crisis, careful consideration including an understanding of current drug use and overdose trends is important before implementing such a law.

This study reports on the physician perceptions of the cause and effect of changing opioid prescribing habits and unintended consequences. It is imperative that future work investigate the direct effect of these trends on patient care and outcomes.

## Conclusions

 An investigation into the impact of SB 273 on prescribing practices amongst WV prescribers revealed broader prescribing changes resulting from fear of disciplinary actions which were then exacerbated by the law implementation. Codification of restrictive prescribing practices into law exacerbated that fear and led to shifts in care, forced tapering and opioid under-prescribing. Prescribers report concern that taking care of patients on opioid medication would put their own careers at risk. A wholistic and patient-centered approach should be taken by legislative and disciplinary bodies to ensure care gaps are addressed when disciplinary actions are taken against prescribers or new legislation is passed.

## Data Availability

Data used for this study can be accessed upon request from the Principal Investigator (Dr. Cara Sedney) at csedney@hsc.wvu.edu.

## Abbreviations

ARIMA: Autoregressive integrated moving average
CDC: The Centers for Disease Control and Prevention
COREQ: COnsolidated criteria for REporting Qualitative research
DEA: Drug Enforcement Administration
PCP: Primary care physician
PDMP: Prescription drug monitoring program
SB 273: Senate Bill 273
SP: Specialists
SRQR: Standards for Reporting Qualitative Research
WVPBRN: West Virginia Practice Based Research Network
